# Clinical outcomes of atezolizumab versus standard-of-care docetaxel with and without ramucirumab in patients with advanced non-small-cell lung cancer who received prior immunotherapy

**DOI:** 10.3389/fonc.2024.1306311

**Published:** 2024-02-07

**Authors:** Shenduo Li, Rami Manochakian, Ruqin Chen, Jaydeepbhai Patel, Jyothik Varun Inampudi, Koshiya R. Hiren, Yujie Zhao, Yanyan Lou

**Affiliations:** ^1^ Division of Hematology and Oncology, Department of Medicine, Mayo Clinic, Jacksonville, FL, United States; ^2^ Department of Internal Medicine, Detroit Medical Center/Wayne State University, Detroit, MI, United States; ^3^ Department of Medicine, Desert Valley Hospital, Victorville, CA, United States

**Keywords:** NSCLC, immunotherapy, atezolizumab, docetaxel, ramucirumab, PD-1/L1

## Abstract

**Background:**

Atezolizumab is superior to docetaxel for patients with advanced non-small-cell lung cancer (NSCLC) who are pretreated with platinum-based chemotherapy based on the POPLAR and OAK trials. However, patients who received prior immunotherapy were excluded from these trials. The standard of care second-line therapy for these patients remains to be docetaxel with or without ramucirumab. The efficacy and safety of atezolizumab as a subsequent therapy in immunotherapy-pretreated patients are unknown.

**Methods:**

We conducted a retrospective study of all patients with locally advanced or metastatic NSCLC who were pretreated with immunotherapy at Mayo Clinic Jacksonville and Rochester from 2016 to 2022. Patients who received subsequent therapy of atezolizumab alone (Atezo), docetaxel (Doce), or docetaxel + ramucirumab (Doce+Ram) were included.

**Results:**

In this cohort of 165 patients, 12.7% (n=21), 49.1% (n=81), and 38.2% (n=63) patients received subsequent Atezo, Doce, and Doce+Ram, respectively. 1-year landmark progression-free survival (PFS) were 23.8%, 6.2%, and 3.2% (p=0.006), and 2-year landmark PFS were 14.3%, 0%, and 0% (p<0.0001), in the Atezo, Doce, and Doce+Ram groups, respectively. About 20% patients with positive PD-L1 had durable response to atezolizumab. The Atezo group showed significantly greater overall survival (OS) improvement over Doce group (median OS 17.7 vs. 7.7 months, HR 0.47, 95% CI 0.29 – 0.76, p=0.008), and over Doce+Ram group (median OS 17.7 vs. 8.9 months, HR 0.55, 95% CI 0.32 – 0.95, p=0.047). 4 of 21 (19%) patients in the Atezo group developed immune-related adverse events (irAE).

**Conclusion:**

We observed statistically significant and clinically meaningful overall survival benefits of atezolizumab monotherapy compared with docetaxel +/- ramucirumab in patients with advanced NSCLC who were pretreated with immunotherapy. The survival benefit seems to be mainly from PD-L1 positive patients. Subsequent immunotherapy with Atezolizumab did not increase irAE rate.

## Introduction

Immune checkpoint inhibitors (ICIs), most commonly programmed cell death-1 (PD-1) and programmed cell death-ligand 1 (PD-L1) inhibitors, have revolutionized the treatment and significantly improved the survival of patients with advanced non-small-cell lung cancer (NSCLC). However, for most patients, their tumor cells inevitably become refractory to treatment over time, resulting in disease progression or recurrence. Subsequent systemic therapy options for patients whose disease progressed on ICI are limited, mostly single agent chemotherapies. Docetaxel is widely used as the preferred subsequent systemic therapy if no actionable driver mutations exist but often has limited survival benefit with reported OS of 6.0-9.1 months (1–4). Ramucirumab, a monoclonal antibody targeting vascular endothelial growth factor (VEGF) receptors, was tested in combination with docetaxel in the phase 3 REVEL clinical trial (3). It was shown to have a 1.4-month improvement in overall survival over docetaxel alone in patients with NSCLC who were pre-treated with platinum-based therapy. Tolerability was a concern as more than 70% of patients experienced grade 3 or higher adverse events (3). In the era of immunotherapy, the efficacy of ramucirumab plus docetaxel was evaluated in a retrospective study where 288 patients who had received previous chemo-immunotherapy were subsequently treated with ramucirumab plus docetaxel. The median PFS and median OS were 4.1 months and 11.6 months, respectively (5).

Atezolizumab is a monoclonal antibody inhibiting PD-L1. It showed improved overall survival (median OS 12.6-13.3 months) compared to docetaxel alone in NSCLC patients who received platinum-based chemotherapy, as demonstrated in the POPLAR and OAK trials (6, 7). Of note, patients who received prior PD-1/PD-L1 inhibitors were excluded from both trials. Sequential use of PD-1/PD-L1 inhibitors has not been adequately assessed in clinical trials, and its efficacy and safety in lung cancer are largely unknown. Few studies consisting of small-size cohorts and case series have been published (8–11). All of them were single-arm studies and did not include control groups of docetaxel with or without ramucirumab for comparison.

Here we conducted a retrospective cohort study including patients with locally advanced or metastatic NSCLC who were pretreated with immunotherapy and received subsequent atezolizumab, docetaxel, or docetaxel plus ramucirumab. We compared the survival outcomes between these three regimens and evaluated the safety and adverse events of ICI rechallenge with atezolizumab in NSCLC patients who received prior PD-1/PD-L1 inhibitors.

## Methods

### Study design and patients

This is a single-institution retrospective study conducted at Mayo Clinic Cancer Center. Patients who were diagnosed with advanced NSCLC and received care at Mayo Clinic Jacksonville and Rochester campuses from 1/1/2016 to 12/31/2022 were screened. Patients were included if they met the following criteria: 1) diagnosed with stage III or stage IV NSCLC, not amendable by localized therapy and had received immunotherapy with a PD-1 or PD-L1 inhibitor; 2) discontinued immunotherapy due to disease progression or adverse events; 3) received subsequent therapy of atezolizumab alone (Atezo), docetaxel (Doce), or docetaxel plus ramucirumab (Doce+Ram). Patients were excluded if they had received maintenance durvalumab after concurrent chemoradiotherapy without subsequent PD-1/PD-L1 inhibitors, or if they lost follow up before the first follow-up visit at our institution.

### Data collection

Data were manually abstracted from the medical chart of each patient, including demographics, pathological diagnosis and staging, treatments, radiographic assessments, biomarkers, and survival status. Categorical variables were summarized as frequency (percentage) and continuous variables were reported as median (range). Patients were grouped based on subsequent therapy (Atezo, Doce, or Doce+Ram). All patients were stratified according to PD-L1 status.

### Outcomes

PFS was defined as the duration from the first dose of subsequent therapy to the date of first radiographic evidence of disease progression, or death of any reason (if occurred before disease progression), or the last follow-up date (if lost follow up before disease progression), or the date cutoff date (if no disease progression). OS was defined as the duration from the first dose of subsequent therapy to death of any reason, or the date that last known to be alive (if lost follow up), or the data cutoff date (if still alive). The data cutoff date was 8/1/2023. Previous immunotherapy best response was defined as the best response from the start of treatment until disease progression based on radiographic assessment, and was categorized into complete response, partial response, stable disease, or disease progression. Adverse events were graded according to the National Cancer Institute Common Terminology Criteria for Adverse Events (CTCAE) version 5.0.

### Statistical analysis

Differences in baseline characteristics among groups were compared by χ^2^ test, Fisher’s exact test, or one-way ANOVA test. Survival analysis was performed by Kaplan-Meier method. The differences between groups were compared using the log-rank test. The correlation of previous ICI response to PFS of atezolizumab was done by Cox proportional hazards regression. All comparisons were two-tailed, with p<0.05 considered significant. The analysis was performed using GraphPad Prism 9.5.0 software and SAS software.

## Results

We screened 646 patients who were previously treated with ICI at Mayo Clinic Jacksonville and Rochester from 2016 to 2022. After applying above inclusion/exclusion criteria, 165 patients were included in this study, and divided into three groups based on subsequent therapies: atezolizumab alone (Atezo, n=21), docetaxel (Doce, n=81), or docetaxel plus ramucirumab (Doce+Ram, n=63). Patients’ demographic characteristics were shown in **Table 1**.

**Table 1:** Baseline characteristics of ICI-pretreated NSCLC patients who received atezolizumab, docetaxel, or docetaxel + ramucirumab.

Characteristic	Atezolizumab N=21	Docetaxel N=81	Doce + Ram N=63	p value
Sex				0.51
Male	(43%)	43 (53%)	28 (44%)	
Female	12 (57%)	38 (47%)	35 (56%)	
Age-year				0.11
Median	73	65	69	
Range	45-89	35-92	38-81	
ECOG PS				0.07
0	3 (14%)	(17%)	15 (24%)	
1	10(48%)	47 758%)	38 (60%)	
2 and above	7 (33%)	18 (22%)	(8%)	
Histology				0.07
Adenocarcinoma	13 (62%)	64 (79%)	51 (79%)	
Squamous	6 (39%)	17 (21%)	10 (16%)	
Others	2 (10%)	0 (0%)	2 (3%)	
Prior ICI regimen				0.24
Pembrolizumab	17 (81%)	73 (90%)	56 (89%)	
Nivolumab	4 (19%)	4 (5%)	5 (8%)	
Others	0 (0%)	4(5%)	2 (3%)	
With/without chemo				<0.001***
ICI + chemo	7(33%)	58 (72%)	49 (78%)	
ICI alone	14 (67%)	23 (28%)	14 (22%)	
Lines of prior therapies				0.46
Median	2	2	2	
Range	1-8	1-5	1-5	
Prior targeted therapies				0.76
Yes	2 (10%)	11 (14%)	10 (16%)	
No	19(90%)	70 (86%)	53 (84%)	
PD-L1 status				0.02*
0	6(29%)	27 (33%)	19 (30%)	
1-49%	(14%)	33 (41%)	16 (25%)	
>50%	11(52%)	14 (17%)	19 (30%)	
Not available	1 (5%)	7 (9%)	9 (14%)	

ECOG, Eastern Cooperative Oncology Group; PS, performance status; ICI, immune checkpoint inhibitor; PD-L1, programmed cell death-ligand 1; Doce, docetaxel; Ram, ramucirumab; * denotes p ≤ 0.05; *** denotes p ≤ 0.001.

In this cohort of 165 patients, 52% were female. The median age was 66 (35 – 92). Most patients (58%) had Eastern Cooperative Oncology Group (ECOG) performance status 1. The histology was predominantly adenocarcinoma (78%). The median number of prior therapies was 2 (1 – 8). Pembrolizumab was the most used prior ICI (87%), followed by nivolumab (8%). 14% patients received prior targeted therapy. 90% patients had PD-L1 status available. Across the three groups, most baseline characteristics were similar. Compared with the other two groups, the Atezo group contained higher percentage of PD-L1 high expression, and more patients who received prior immunotherapy as monotherapy rather than in combination with chemotherapy. The Atezo group tended to have more elderly patients and higher ECOG scale, but not statistically different from the other two groups. These differences could possibly be attributed to treating clinician’s choice of treatment based on the evidence that elderly and fragile patients with high PD-L1 expression may have better efficacy and tolerability of immunotherapy than chemotherapy (12). At the data cutoff date, the median follow-up time is 27.7 months, 127 patients had died, 18 patients had lost follow up, and 20 patients were alive.

**Figure 1:**
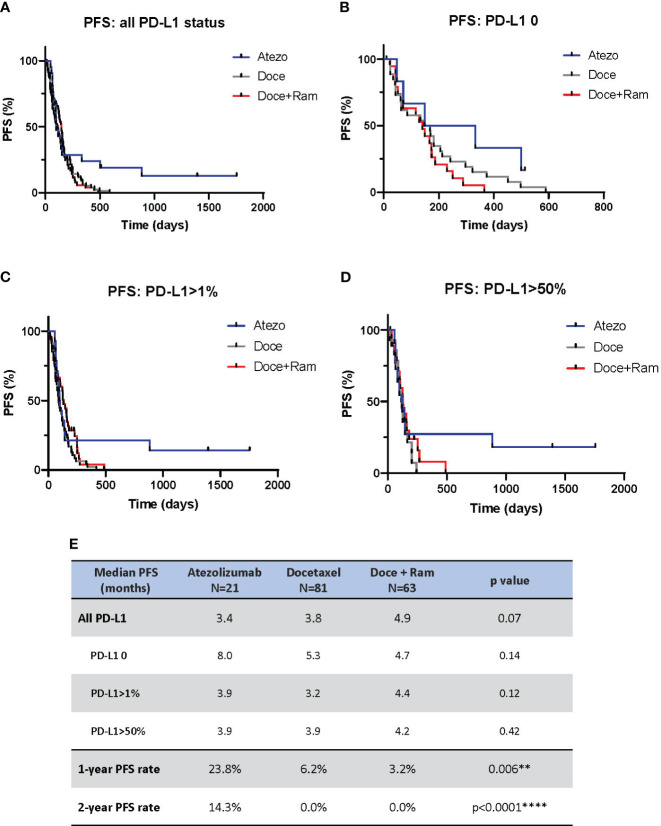
Progression-free survival (PFS) comparison by Kaplan-Meier curves for all patients **(A)**, and stratified by PD-L1 negative **(B)**, PD-L1 positive **(C)**, and PD-L1 high **(D)**. Summary of median PFS and 1- and 2-year landmark PFS across three groups **(E)**. Atezo, atezolizumab; Doce, docetaxel; Ram, ramucirumab; PD-L1, programmed cell death-ligand 1; HR, hazard ratio; Cl, confidence interval. ** denotes p ≤ 0.01; **** denotes p ≤ 0.0001.

PFS analysis is shown in **Figure 1**. We observed no statistically different median PFS across three groups (3.4 vs. 3.8 vs. 4.9 months in Atezo, Doce, and Doce+Ram groups, respectively, p=0.07). However, a prominent percentage of patients in the Atezo group appear to have long-term PFS benefits, demonstrated by 1-year landmark PFS of 23.8%, 6.2%, and 3.2% (p=0.006), and 2-year landmark PFS of 14.3%, 0%, and 0% (p<0.0001) in the Atezo, Doce, and Doce+Ram groups, respectively. In subgroup analysis stratified by PD-L1 level, no significant difference in median PFS was observed across treatments in each PD-L1 subgroup. However, about 20% patients with positive PD-L1 appeared to have durable response to atezolizumab (**Figures 1C, D**).

**Figure 2:**
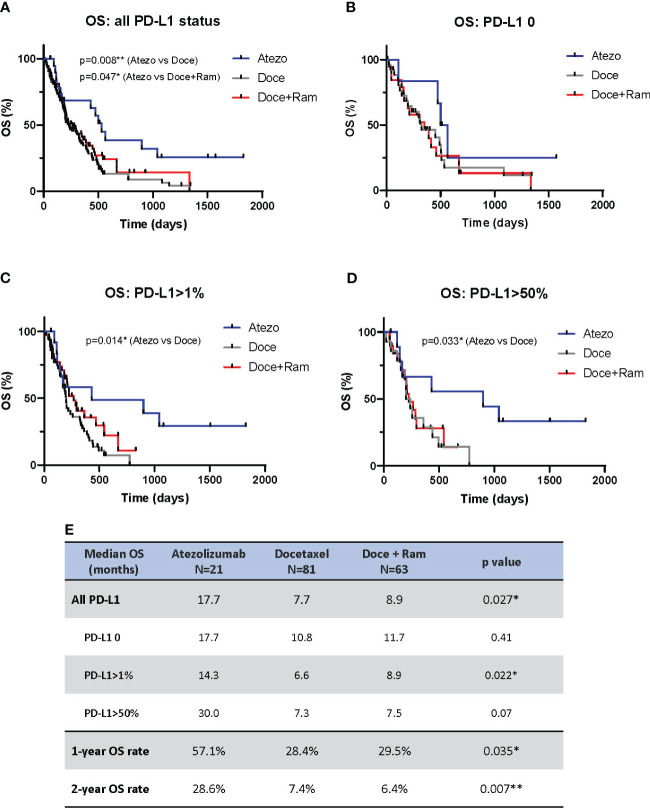
Overall survival (OS) comparison by Kaplan-Meier curves for all patients **(A)**, and stratified by PD-L1 negative **(B)**, PD-L1 positive **(C)**, and PD-L1 high **(D)**. Summary of median OS and 1- and 2-year landmark OS across three groups **(E)**. Atezo, atezolizumab; Doce, docetaxel; Ram, ramucirumab; PD-L1, programmed cell death-ligand 1; HR, hazard ratio; Cl, confidence interval. * denotes p ≤ 0.05; ** denotes p ≤ 0.01.

**Figure 2** showed the results of OS analysis. We observed statistical difference in median OS across three treatments (17.7 vs. 7.7 vs. 8.9 months in Atezo, Doce, and Doce+Ram groups respectively, p=0.027). Atezolizumab showed significantly prolonged OS compared to docetaxel (median OS 17.7 vs. 7.7 months, HR 0.47, 95% CI 0.29 – 0.76, p=0.008) and docetaxel plus ramucirumab (median OS 17.7 vs. 8.9 months, HR 0.55, 95% CI 0.32 – 0.95, p=0.047). 1- and 2-year landmark OS were also much higher in Atezo group (1-year OS rates of 57.1%, 28.4%, and 29.5% [p=0.035], and 2-year PFS rates of 28.6%, 7.4%, and 7.4% [p=0.007] in the Atezo, Doce, and Doce+Ram groups, respectively). In terms of PD-L1 levels, Atezolizumab demonstrated significantly prolonged OS compared with docetaxel in PD-L1-positive subgroup (median PFS 14.3 vs. 6.6 months, HR 0.43, 95% CI 0.24 – 0.78, p=0.014) and in PD-L1-high subgroup (median PFS 30.0 vs. 7.3 months, HR 0.39, 95% CI 0.16 – 0.97, p=0.033). When compared with docetaxel plus ramucirumab, atezolizumab showed numerically longer median OS in all PD-L1 subgroups. The OS benefit appears greater in PD-L1 high population.

**Figure 3:**
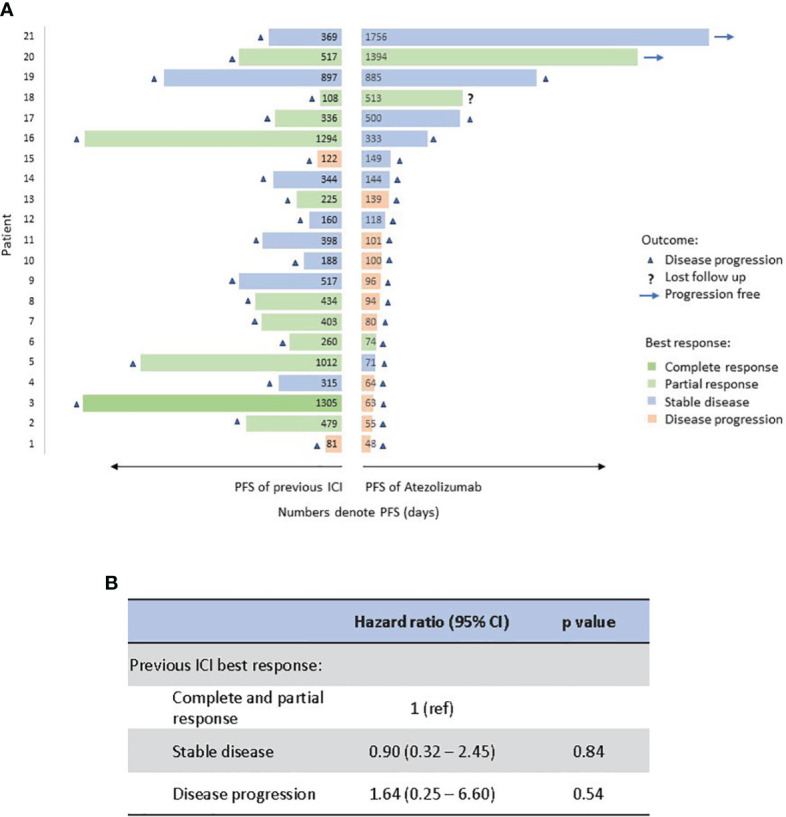
Comparison of PFS (in length) and best response (in color) between atezolizumab and previous immunotherapy for each patient. Numbers represent PFS in days **(A)**. Correlation of previous ICI response to PFS of atezolizumab by Cox proportional hazards regression analysis **(B)**. PFS, progression-free survival; Atezo, atezolizumab; ICI, immune checkpoint inhibitor.

We further compared PFS and best treatment response of atezolizumab to those of prior immunotherapy for each patient in the Atezo group (**Figure 3A**). During previous ICI treatment, one patient had complete response, 10 patients had partial response, 8 patients had stable disease, and 2 patients had disease progression. 17 patients discontinued previous ICI due to eventual disease progression, and 4 patients discontinued due to adverse events but all had disease progression subsequently. During atezolizumab treatment, the median of PFS to atezo is 3.4 months, 8 patients remained as stable disease, 3 patients had partial response and 10 patients had cancer progression. Best treatment response to prior immunotherapy does not correlate with PFS of subsequent atezolizumab, though the patient number is small to derive statistical difference (**Figure 3B**).

4 of 21 (19%) patients in the Atezo group developed immune-related adverse events (irAE) (**Table 2**). Two patients were grade 3. One patient had possible grade 4 pneumonitis. No grade 5 event. Additionally, one patient stopped atezolizumab due to grade 2 anemia, which was later considered to be caused by concurrent chemotherapy. One patient stopped atezolizumab after grade 3 colitis likely of infectious etiology rather than immune related.

**Table 2:** Adverse events occurred in patients receiving atezolizumab.

Patient	irAE	Grade
#1	mucositis	2
#2	elevated LFT	3
#3	adrenal insuff.	3
#4	pneumonitis	4
#5	anemia*	2
#6	colitis*	3

Grade is based on Common Terminology Criteria for Adverse Events (CTCAE) 5.0. irAE, immune-related adverse event; LFT, liver function test.

*likely non-immune related.

## Discussion

There is an unmet need for an effective subsequent therapy for patients with NSCLC without targetable mutation and disease progressed on chemoimmunotherapy. In 2014, ramucirumab was approved by the U.S. Food and Drug Administration (FDA) to be used in combination with docetaxel as a subsequent therapy before the immunotherapy era. Recently, as the immunotherapy is widely used in first-line setting, several retrospective studies re-evaluated the efficacy of adding ramucirumab to docetaxel, and the additional survival benefit appears only modest (5, 13). In our single-institution retrospective study, we observed statistically significant and clinically meaningful OS advantage of atezolizumab monotherapy over standard-of-care docetaxel with or without ramucirumab. Although median PFS was not increased in Atezo group (3.4 months) compared with Doce (3.8 months) or Doce+Ram (4.9 months), the 1-year and 2-year landmark PFS are significantly prolonged in the Atezo group in compared to other two groups. This is consistent with many other clinical studies that landmark PFS is likely better reflecting the clinical benefits of immune checkpoints due to durable response in selective patients. In addition, we also observed significantly improved OS (17.7 vs. 7.7 vs. 8.9 months) in the Atezo group in compared to other two groups despite small sample size. This discordance between PFS and OS is consistent with previous studies that showed atezolizumab and other ICIs may have delayed anti-tumor effect that lasts beyond treatment period (6, 14, 15). Median PFS correlates poorly with median OS and may underestimate the clinical benefits of immunotherapy (16, 17). This phenomenon can possibly be explained by the initial tumor volume increase due to immune infiltration and delayed antitumor immune activation (6). Nevertheless, overall survival is still considered the best criterion and gold standard for evaluating treatment efficacy in lung cancer (18).

PD-L1 expression is a pivotal although imperfect biomarker to predict ICI efficacy in NSCLC. In our study, we observed greater OS advantage in PD-L1 high (>50%) patients with a median OS of 30 months. The survival curve separated and plateaued much earlier apart from the Doce+/-Ram groups when compared with PD-L1 low or negative subgroups. Our observation is consistent with findings in several large prospective studies including IMpower110, Impower150, and OAK trials that PD-L1 high expression is associated with greater survival benefit in response to atezolizumab (6, 19, 20). In our cohort, there were more PD-L1 high patients in the Atezo group, which may potentially correlate with the prolonged survival outcomes compared with the other two treatment groups. Other biomarkers, such as tumor mutation burden (TMB) and microsatellite instability (MSI), were reportedly to correlate with ICI efficacy (21, 22). However, only limited number of patients in our cohort had TMB or MSI information available, insufficient for meaningful analysis.

Sequential use or rechallenge of PD-1/PD-L1 inhibitors remains controversial in lung cancer treatment. Current NCCN guideline does not recommend subsequent use of another PD-1/PD-L1 inhibitor after disease progression on first-line PD-1/PD-L1 inhibitor (23). Low efficacy is a major concern. As all PD-1/PD-L1 inhibitors target similar pathway, resistance to one ICI may lead to class resistance and treatment response to a second ICI is likely low (8, 9, 24). Another concern is increased toxicity. One study showed subsequent treatment of PD-1 and PD-L1 inhibitors can lead to fulminant cardiotoxicity (25). To challenge this notion, a recent phase II randomized study demonstrated OS benefit of pembrolizumab in combination with ramucirumab over standard of care (mainly docetaxel and ramucirumab) in patients whose disease progressed on chemoimmunotherapy (median OS 14.5 vs. 11.6 months, HR 0.69, 80% CI 0.51 to 0.92, p=0.05). irAE incidence was not higher than what’s expected for ICIs (26). To our knowledge, so far, no prospective studies have evaluated PD-1/PD-L1 inhibitor monotherapy after prior immunotherapy in advanced NSCLC. Our study suggested that subsequent use of atezolizumab alone may confer prominent clinical benefits and overcome immunotherapy resistance in those patients. Toxicity appears to be acceptable (irAE rate 19%, 4/21 patients).

PD-1 inhibitor and PD-L1 inhibitor work on the same PD-1/L1 axis but slightly different. PD-1 inhibitor blocks both PD-L1 and PD-L2, whereas PD-L1 inhibitor also blocks the binding to CD80 which releases CTLA-4-mediated anti-tumor immunity (27, 28). In our atezolizumab group, all patients had experienced disease progression on a PD-1 inhibitor either pembrolizumab or nivolumab. It is unclear whether PD-L1 inhibitor such as atezolizumab may overcome the immunotherapy resistance through alternative pathways. Further, it is unknown whether the survival benefit observed in our study is limited to the specific PD-1-then-PD-L1 blockade sequential treatment strategy.

Previous study showed that immunotherapy rechallenge after prior nivolumab treatment resulted in better survival in patients with a longer duration of initial nivolumab treatment (29). Therefore, we examined whether treatment response to previous ICI can predict PFS of subsequent atezolizumab monotherapy and found no correlation. In our study, we did not identify a reliable factor or biomarker that correlates or predicts the efficacy of subsequent atezolizumab therapy. Finding effective predictive biomarkers to select patients likely to benefit from immunotherapy still remains a prevalent challenge worldwide.

Our study has several limitations. It was a single-institution experience with a relatively small cohort. Atezolizumab alone after prior use of immunotherapy is not widely used nationwide which makes the expansion of cohort difficult. For example, we did not find an eligible patient to be included in Mayo Clinic Arizona campus. The small number of patients in the atezolizumab group, limited the power of statistical analysis, especially subgroup analysis. The study was retrospective, which means the treatment strategy was not randomized into all three groups. In addition, the imbalanced clinical features among the groups were also noticed in our study, partially due to overall small sample size. For example, squamous cell carcinoma represented 39% of patients in the Atezolizumab arm versus 21% in Docetaxel arm, although the difference was not statistically significant. Similarly, much more patients received prior chemotherapy in combination with immunotherapy in the docetaxel with or without ramucirumab arm in comparison to Atezolizumab arm, highlighting that potential factors, such as age, ECOG status, PD-L1 expression, histology and response to previous ICI, may have influenced clinician’s treatment choice. Recently, a pooled analysis of KEYNOTE-010, KEYNOTE-024, and KEYNOTE-042 studies showed pembrolizumab monotherapy is superior to chemotherapy in elderly patients with positive PD-L1 (12). Our study showed that similar population of patients may also benefit from subsequent atezolizumab monotherapy. However, we do not know why a subset of ICI-pretreated patients achieved long term response and survival advantage on atezolizumab. Further studies are needed to identify better predictive factors or establish an algorithmic model to select patients who will benefit from sequential immunotherapy.

In conclusion, we observed statistically significant and clinically meaningful survival benefits of atezolizumab monotherapy compared with docetaxel +/- ramucirumab in patients with advanced NSCLC who were pretreated with ICI. The OS benefits of atezolizumab over docetaxel was greater in PDL1>1% and PD-L1>50% subgroups. Our study challenged the current treatment guideline by showing subsequent use of immunotherapy alone may be beneficial to ICI-pretreated NSCLC patients, particularly PD-L1 >1% and PD-L1>50% patients. Further multi-institutional retrospective study is needed to verify these results. Prospective clinical trials are in demand to evaluate the clinical efficacy of immunotherapy rechallenge as a new strategy for ICI-pretreated NSCLC patients. Additionally, whether immune checkpoint inhibitors other than atezolizumab can be subsequently used in this setting. Finally, other immune-based therapeutic strategies, such as chimeric antigen receptor-T-cell therapy, bispecific T cell engagers, cancer vaccines, should be explored for the goal of benefiting NSCLC patients who suffer from disease progression after first-line chemoimmunotherapy.

## Data availability statement

The original contributions presented in the study are included in the article/supplementary material. Further inquiries can be directed to the corresponding author.

## Ethics statement

The studies involving humans were approved by Mayo Clinic institutional review board. The studies were conducted in accordance with the local legislation and institutional requirements. The ethics committee/institutional review board waived the requirement of written informed consent for participation from the participants or the participants’ legal guardians/next of kin because Based on the nature of retrospective study.

## Author contributions

SL: Conceptualization, Data curation, Formal Analysis, Investigation, Methodology, Writing – original draft, Writing – review & editing. RM: Conceptualization, Investigation, Supervision, Writing – review & editing. RC: Formal Analysis, Investigation, Methodology, Writing – review & editing. JP: Data curation, Writing – review & editing. JI: Data curation, Writing – review & editing. KH: Data curation, Writing – review & editing. YZ: Writing – review & editing. YL: Conceptualization, Project administration, Resources, Supervision, Writing – review & editing.
